# Clinical characteristics, visceral involvement, and mortality in at-risk or early diffuse systemic sclerosis: a longitudinal analysis of an observational prospective multicenter US cohort

**DOI:** 10.1186/s13075-021-02548-1

**Published:** 2021-06-14

**Authors:** Sara Jaafar, Alain Lescoat, Suiyuan Huang, Jessica Gordon, Monique Hinchcliff, Ami A. Shah, Shervin Assassi, Robyn Domsic, Elana J. Bernstein, Virginia Steen, Sabrina Elliott, Faye Hant, Flavia V. Castelino, Victoria K. Shanmugam, Chase Correia, John Varga, Vivek Nagaraja, David Roofeh, Tracy Frech, Dinesh Khanna

**Affiliations:** 1 Division of Rheumatology and Scleroderma Program, Department of Internal Medicine, University of Michigan, Suite 7C27 300 North Ingalls Street, SPC 5422, Ann Arbor, MI 48109 USA; 2 Department of Internal Medicine and Clinical Immunology, CHU Rennes, Univ Rennes, Rennes, France; 3 University of Rennes, CHU Rennes, Inserm, EHESP, Irset (Institut de recherche en santé, environnement et travail, Rennes, France; 4 School of Public Health, University of Michigan, Ann Arbor, USA; 5 Department of Medicine, Hospital for Special Surgery, New York, NY USA; 6 Department of Medicine, Northwestern University, Chicago, IL USA; 7 Department of Medicine, Johns Hopkins University, Baltimore, MD USA; 8 Department of Medicine, University of Texas Health Science Center, Houston, TX USA; 9 Department of Medicine, University of Pittsburgh Medical Center, Pittsburgh, PA USA; 10 Department of Medicine, Columbia University Irving Medical Center, New York, NY USA; 11 Department of Medicine, Georgetown University, Washington, DC USA; 12 Department of Medicine, Medical University of South Carolina, Charleston, SC USA; 13 Department of Medicine, Harvard University, Boston, MA USA; 14 Department of Medicine, George Washington University, Washington, DC USA; 15 Department of Medicine, University of Utah, Salt Lake City, UT USA

**Keywords:** Systemic sclerosis, Scleroderma, Diffuse cutaneous systemic sclerosis, Mortality, Survival, Interstitial lung disease

## Abstract

**Background:**

Early diffuse cutaneous systemic sclerosis (dcSSc) has the highest case fatality among rheumatic diseases. We report baseline characteristics, current immunosuppressive therapies, progression of skin and internal organ involvement, and mortality in a multicenter prospective cohort from the United States (US) of America.

**Methods:**

We performed a longitudinal analysis of participants from 12 US centers, from April 2012 to July 2020. All participants had early dcSSc or were at-risk for dcSSc, with ≤2 years since the first non-Raynaud’s phenomenon (RP) symptom.

**Results:**

Three hundred one patients were included with a baseline median disease duration of 1.2 years since RP and a mean modified skin score of 21.1 units. At baseline, 263 (87.3%) had definite dcSSc and 38 (12.7%) were classified as at-risk; 112 (49.6%) patients were positive for anti-RNA polymerase III antibodies. The median follow-up duration was 24.5 months (IQR = 10.3–40.7 months). One hundred ninety (63.1%) participants were treated with an immunosuppressive therapy, of which mycophenolate mofetil was most used at baseline and follow-up. Of 38 who were classified as at-risk at baseline, 27 (71%) went on to develop dcSSc; these patients were characterized by higher baseline mean HAQ-DI (0.8 versus 0.4, p = 0.05) and higher baseline mRSS (8.8 versus 4.4, p < 0.01) in comparison with those who remained as limited cutaneous SSc. In the overall cohort, 48 participants (21.1%) had clinically significant worsening of skin fibrosis, mainly occurring in the first year of follow-up; 41 (23.3%) had an absolute forced vital capacity decline of ≥10%. Twenty participants (6.6%) died, of which 18 died in the first 3 years of follow-up. Cardiac involvement (33.3%), gastrointestinal dysmotility (22.2%), and progressive interstitial lung disease (ILD) (16.7%) were the main causes of death.

**Conclusion:**

This US cohort highlights the management of early SSc in the current era, demonstrating progression of skin and lung involvement despite immunosuppressive therapy and high mortality due to cardiac involvement.

## Background

Systemic sclerosis (SSc or scleroderma) is a rheumatological disorder characterized by occlusive microangiopathy associated with fibrotic features, such as skin or lung fibrosis, and the presence of autoimmune markers including specific antibodies [1]. Among all rheumatic disorders, SSc has the highest case-specific mortality with a detrimental impact on quality of life. According to the extent of skin involvement, two main subsets of SSc are described: limited cutaneous SSc (lcSSc) and diffuse cutaneous SSc (dcSSc); dcSSc accounts for almost one-third of all patients with SSc [2]. dcSSc is considered the most severe subset due to lower survival rate, higher overall progression, and severity of skin and visceral involvement. Interstitial lung disease (ILD) and cardiac involvement (including pulmonary arterial hypertension) are currently considered as the leading causes of SSc-associated mortality [1].

Major changes have been made in the management of SSc in recent years, based on the results of randomized control trials (RCTs) demonstrating the positive impact of immunosuppressive drugs and autologous hematopoietic stem cell transplant on visceral involvement [3–5]. Early management and the use of immunosuppressive therapies have thus become the cornerstones for the evidence-based management of patients with dcSSc [6]. The clinical impact of these modifications in the last decade and their effective implementation in routine medical care is still to be precisely determined, especially in patients with very early dcSSc, defined by less than 2 years since the onset of the first non-Raynaud’s phenomenon (RP) symptom.

Considering the rarity of early dcSSc, current knowledge about the natural history of dcSSc is mainly based upon nationwide or international observational cohorts [7–9]. These cohorts do not usually focus on early dcSSc, but on dcSSc regardless of the disease duration or on early dcSSc defined by a disease duration of less than 5 years. The placebo arms of RCTs are also informative, but these patients are highly selected due to inclusion criteria or a primary objective based on single organ involvement; these cohorts may not be representative of the overall population of early dcSSc patients in the current era [10, 11]. Cohorts from single centers have also provided insight, but the rarity of early dcSSc leads to long inclusion periods to obtain a significant sample size. Moreover, center bias exists and single-center studies may not reflect a nationwide practice for the management of early dcSSc [12, 13]. Therefore, multicenter nationwide observational studies dedicated to early dcSSc are needed [6].

The first years following the onset of RP have been identified as a window of opportunity for the management of dcSSc. At the very beginning of the disease, dcSSc patients may not have a diffuse cutaneous involvement per se and may initially present with limited skin involvement. The natural history of this subgroup of patients with initial limited skin involvement but at high risk of dcSSc based on the presence of clinical parameters such as tendon friction rubs (TFR) or immunological features such as positivity for anti-RNA polymerase III or anti-Scl70 antibodies is still to be further described [14].

A precise overview of the clinical characteristics and treatments of patients with early or at-risk dcSSc based on real-life data in present standard of care may help improve the design of such future clinical trials. The current multicenter longitudinal prospective observational PRESS cohort provides a unique opportunity to comprehensively assess the baseline characteristics, treatment patterns, and progression of early at-risk or early dcSSc in the current era. The present study has the following objectives: to assess the baseline characteristics of patients with early at-risk or definite dcSSc, describe current immunosuppressive therapies used for the management of this subset, and assess progression of early dcSSc, as it relates to skin and internal organ involvement, and mortality.

## Methods

### Study participants

Adult participants (≥18 years) included in this study were from the observational longitudinal multicenter national Prospective Registry of Early Systemic Sclerosis (PRESS) registry that recruited participants with a diagnosis of early (defined as ≤2 years since the first non-RP symptom [15]) dcSSc [2], or at-risk for dcSSc, defined as patients with swollen hands or sclerodactyly associated with the presence of anti-topoisomerase I or anti-RNA polymerase III antibodies, and/or presence of tendon friction rubs, but with still limited skin involvement in this early phase of the disease [14, 16].

All participants provided written consent to participate in this IRB-approved registry that started in April 2012. Participating sites in this national US registry included the University of Michigan, Columbia University, Harvard University, George Washington University, Georgetown University, Hospital for Special Surgery, John Hopkins University, Medical University of South Carolina, Northwestern University, University of Pittsburgh, University of Texas at Houston Health Science Center, and University of Utah. The data management was housed at the University of Michigan, and all data for patients registered in the PRESS registry as of July 2020 were exported for the present analysis.

### Outcomes and follow-up

Data was captured at baseline and every 6 months (±3 months) when a participant presented to the clinic for a standard of care visit. Case report forms captured age, gender, race, past and current use of immunosuppressive medications, scleroderma-specific antibodies (done locally as part of clinical care), modified Rodnan skin score (mRSS), and any standard of care assessments such as pulmonary function test (PFT), right heart catheterization (RHC), transthoracic echocardiogram (TTE), or chest imaging (chest X-ray or high-resolution computed tomography (HRCT)). Physicians completed a vascular, cardiopulmonary, gastrointestinal, renal, and vascular standardized assessment at the specified time points. Participants also completed the HAQ-DI questionnaire. Pulmonary hypertension (PH) was defined by a mPAP ≥ 25 mmHg on RHC, in accordance with international guidelines [17]. The date of the first non-RP symptom and the date of RP onset were retrospectively recorded at inclusion to define disease duration.

Concerning skin and visceral progression during follow-up, clinically significant worsening of skin disease was defined as an absolute increase of mRSS ≥ 5 units or ≥ 25% as compared to baseline mRSS [8]; significant functional progression of ILD was defined as an absolute FVC decline of ≥10% as compared to baseline FVC during the whole course of the study [18]. Left ventricular ejection fraction (LVEF) ≤ 45% on TTE at baseline and follow-up was specifically considered for cardiac involvement. Patients’ vital status and cause of death, confirmed from medical records or death certificates, were captured as well.

### Statistical analysis

We reported mean and standard deviation (SD) for quantitative variables with Gaussian distribution and reported median and first and third quartile for quantitative variables with non-Gaussian distribution. We checked the normality for numerical variables via a descriptive graph—histogram, and a theory-driven graph—QQ plot. Count and percent were reported for categorical variables for the whole cohort. Regarding immunosuppressive medication intake, we calculated the mean (SD) of baseline dose for each medication. We also calculated the average dose intake for each medication during the study by summing up the time of intake of each dose level for all participants, multiplying this dose by the corresponding sum of time for each participant, dividing the product by the overall time of intake to get an average dose for each participant, and then getting mean (SD) from the average dose from those whoever took the medication during the study. We explored percentages of mRSS worsening and FVC worsening as previously defined. Worsening was counted among those who had baseline and at least one follow-up measurement. We also explored PH, LVEF ≤45%, scleroderma renal crisis (SRC), and all-cause mortality. For PH, LVEF ≤45%, and renal crisis, we reported counts and percentages in two parts: (1) events before/at baseline and (2) events during follow-up among those who did not have events before/at baseline. We reported PH, SRC, and mortality for the whole cohort and reported LVEF ≤45% for those who had TTE. All analyses described above were conducted in SAS (version 9.4). Additionally, we did time-to-event analysis for mRSS worsening, FVC worsening, and all-cause mortality, by plotting cumulative event curves. Figures were plotted via R package “survival” and “survminer” (R version 4.0.2).

## Results

### Baseline characteristics and demographics

The cohort consisted of 301 participants at baseline with a median follow-up of 24.5 months (IQR = 10.3–40.7 months). The mean (SD) age of the cohort was 50.7 (13.8) years, 70.1% were female, 73.8% were White, median disease duration was 1.2 years (25th–75th range 0.7, 2.0) since RP vs. 1.1 (0.7, 1.6) years since the first non-RP symptom, and 45.2% had puffy hands or fingers as the first non-RP symptom (Table 1). The mean (SD) baseline mRSS was 21.1 (10.2). Seventy-two participants (28.9%) were positive for the anti-topoisomerase I antibody, 49.6% were positive for anti-RNA polymerase III antibody, and 89% were ANA positive. 53.6% of the subjects had evidence of ILD on their baseline HRCT and the PFTs revealed a mean FVC (% predicted) of 81.0 (18.6; n = 256) and DL_CO_ (% predicted) of 70.6 (24.6, n = 243). Sixteen (5.3%) participants had a history of SRC that occurred before the baseline visit, 27 (13.9%) had a pericardial effusion on baseline TTE, and 5 (1.7%) had a history of PH on RHC (Table 1).

**Table 1:** Baseline PRESS demographic and clinical characteristics in the overall population and according to baseline cutaneous subgroups (n = 301)

Baseline characteristics (***n*** = total available data)^∫^	Overall population ***N*** = 301	Definite dcSSc ***n*** = 263	At risk for dcSSc ***n*** = 38	***P***-value^**£**^
**Demographic data**				
Age (years), mean (±SD), (*n* = 301)	50.7 (±13.8)	51.5 (±13.7)	45.3 (±12.8)	0.0094^≉^
Gender/female, n (%), (*n* = 301)	211 (70.1)	181 (68.8)	30 (78.9)	0.2025^¶^
Race, n (%), (*n* = 301)				
Black	50 (16.6)	46 (17.5)	4 (10.5)	0.5090^§^
White	222 (73.8)	191 (72.6)	31 (81.6)	
Others	24 (7.9)	22 (8.4)	2 (5.3)	
Unknown	5 (1.7)	4 (1.5)	1 (2.6)	
Ethnicity, n (%), (*n* = 301)				
Hispanic	32 (10.6)	28 (10.6)	4 (10.5)	1.0000^§^
Non-Hispanic	264 (87.7)	230 (87.5)	34 (89.5)	
Others/unknown	5 (1.6)	5 (1.9)	0 (0.0)	
Marital status, n (%), (*n* = 301)				
Single	62 (20.6)	55 (20.9)	7 (18.4)	0.5936^§^
Married	202 (67.1)	173 (65.8)	29 (76.3)	
Divorced or widowed	27 (9.0)	25 (9.5)	2 (5.3)	
Others/unknown	10 (3.3)	10 (3.8)	0 (0.0)	
Employment status, n (%), (*n* = 301)				
Full-time	156 (51.8)	131 (49.8)	25 (65.8)	0.0239^¶^
Part-time	15 (5.0)	11 (4.2)	4 (10.5)	
Retired	49 (16.3)	46 (17.5)	3 (7.9)	
Disability/disabled	22 (6.0)	22 (6.8)	0 (0.0)	
Disabled due to scleroderma	18 (7.3)	18 (8.4)	0 (0.0)	
Others^+^	41 (13.6)	35 (13.3)	6 (15.8)	
Smoking status, n (%), (*n* = 301)				
Never	187 (62.1)	157 (59.7)	30 (78.9)	0.0222^¶^
Current or former	114 (37.8)	106 (40.3)	8 (21.1)	
**Clinical data**				
Disease duration (years), mean (±SD), median (IQR)^∫^ since first non-RP symptoms (*n* = 301)	1.2 (±0.7), 1.1 (0.7, 1.6)	1.2 (±0.7), 1.1 (0.7, 1.6)	1.0 (±0.5), 0.9 (0.7, 1.3)	0.1171^€^
Disease duration (years), mean (±SD), median (IQR) since Raynaud’s phenomenon (*n* = 281)	2.5 (±5.0), 1.2 (0.7, 2.0)	2.4 (±4.7), 1.2 (0.7, 2.0)	3.7 (±6.4), 1.3 (0.8, 2.5)	0.4240^€^
Disease duration less than 6 months, n (%), (*n* = 301)	31 (10.3)	27 (10.3)	4 (10.5)	1.0000^§^
First scleroderma symptom, n (%), (*n* = 301)				
Puffy hands or fingers	136 (45.2)	117 (44.5)	19 (50.0)	0.2270^¶^
Dyspnea	12 (4.0)	11 (4.2)	1 (2.6)	
Arthritis	19 (6.3)	18 (6.8)	1 (2.6)	
Reflux	5 (1.7)	4 (1.5)	1 (2.6)	
Raynaud’s phenomenon	64 (21.3)	51 (19.4)	13 (34.2)	
Skin tightening	38 (12.6)	37 (14.1)	1 (2.6)	
DU	4 (1.3)	4 (1.5)	0 (0.0)	
Others*	23 (7.6)	21 (8.0)	2 (5.3)	
Baseline mRSS (*n* = 297), mean (±SD)	21.1 (±10.2)	22.9 (±9.3)	7.4 (±4.8)	<.0001^≉^
Tendon friction rubs, n (%), (*n* = 285)	97 (34.0)	90 (36.3)	7 (18.9)	0.0375^¶^
Active DU, n (%), (*n* = 279)	17 (6.1)	16 (6.6)	1 (2.7)	0.7091^§^
Calcinosis, n (%), (*n* = 281)	20 (7.1)	16 (6.5)	4 (11.4)	0.2905^§^
ILD on HRCT, n (%), (*n* = 239)	128 (53.6)	112 (53.8)	16 (51.6)	0.8161^¶^
FVC (n = 256) (%pred), mean (±SD)	81.0 (±18.6)	79.9 (±18.3)	88.6 (±19.0)	0.0102^≉^
FVC <7 0%, n (%), (*n* = 256)	78 (30.5)	70 (31.5)	8 (23.5)	0.3452^¶^
DLCO (n = 243) (%pred), mean (±SD)	70.6 (±24.6)	69.3 (±23.5)	79.7 (±29.8)	0.0259^≉^
History of PH based on baseline RHC,^+^ n (%), (*n* = 301)	5 (1.7)	4 (1.5)	1 (2.6)	0.4932^§^
Pericardial effusion on first TTE, n (%), (*n* = 194)	27 (13.9)	25 (14.6)	2 (8.7)	0.7474^§^
LVEF of ≤ 45% on first TTE, n (%), (*n* = 138)	3 (2.2)	3 (2.5)	0 (0.0)	1.0000^§^
History of scleroderma renal crisis, n (%), (*n* = 301)	16 (5.3)	15 (5.7)	1 (2.6)	0.7030^§^
HAQ-DI (n = 259), mean (±SD), median (IQR)	1.1 (±0.7), 1.1 (0.5, 1.6)	1.2 (±0.7), 1.1 (0.5, 1.6)	0.7 (±0.6), 0.5 (0.3, 1.0)	0.0003^€^
**Biological data**				
ANA positive, n (%), (*n* = 255)	227 (89.0)	195 (87.8)	32 (97.0)	0.1438^§^
Anti-Topo I (*n* = 249)	72 (28.9)	55 (25.1)	17 (56.7)	0.0004^¶^
Anti-RNA pol III (*n* = 226)	112 (49.6)	102 (51.3)	10 (37.0)	0.1655^¶^
Anti-U3 RNP/fibrillarin (*n* = 64)	1 (1.6)	1 (1.8)	0 (0.0)	1.0000^§^
Anti-centromere (*n* = 212)	6 (2.8)	6 (3.2)	0 (0.0)	1.0000^§^
Anti-Th/To (*n* = 54	7 (13.0)	3 (6.5)	4 (50.0)	0.0063^§^
SSA/anti-RO (*n* = 201)	26 (12.9)	23 (13.0)	3 (12.5)	1.0000^§^
SSB/anti-LA (*n* = 201)	5 (2.5)	4 (2.3)	1 (4.2)	0.4741^§^
Baseline CRP value, mean (±SD), median (IQR) (*n* = 179) mg/dL	2.2 (±3.3), 0.7 (0.4, 2.7)	2.2 (±3.4), 0.7 (0.4, 2.4)	2.3 (±2.7), 0.7 (0.4, 4.3)	0.6330^€^
CRP > ULN (0.6 mg/dL), n (%), (*n* = 179)	98 (54.7)	85 (54.1)	13 (59.1)	0.6622^¶^

*mRSS*, modified Rodnan skin score; *FVC*, forced vital capacity; *DL*_*CO*_, diffusion capacity for carbon monoxide; *RHC*, right heart catheterization; *LVEF*, left ventricular ejection fraction; *TTE*, transthoracic echocardiogram; *ILD*, interstitial lung disease; *Topo I*, topoisomerase I; *RNA pol III*, RNA polymerase III; *CRP*, C-reactive protein; *HAQ-DI*, Health Assessment Questionnaire-Disability Index; *IQR*, inter-quartile range; *SD*, standard deviation; *ULN*, upper limit of normal

*Other first scleroderma symptom includes lower extremity swelling, telangiectasias, wrist and ankle inflammation, joint pain, fatigue, myalgias, Carpal tunnel syndrome, cold and numbness in extremities, pruritis, hypo/hyper-pigmentation, hypertension, cough, and gastrointestinal discomfort

^+^Based on the results of n = 22 RHC on 22 participants

^∫^Data are expressed as n (%) unless otherwise specified; quantitative data without Gaussian distribution are presented as median (IQR) as specified

^£^Comparison between definite dcSSc and high-risk population at baseline

^≉^t-test

^¶^Chi-squared test

^§^Fisher exact test

^€^Wilcoxon rank sum test

The overall cohort was further classified into definite dcSSc and at-risk group at baseline. The mean (SD) age was 51.5 (13.7) vs. 45.3 (12.8) years, median disease duration was 1.1 vs. 0.9 years since the first non-RP symptom, and 44.5% vs. 50.0% had puffy hands or fingers as the first non-RP symptom among the definite dcSSc vs. at-risk group, respectively (Table 1). Among the 38 participants in the at-risk group at baseline, 27 (71%) developed dcSSc at follow-up and 11 remained as limited cutaneous or sine SSc (Table 2). Among the at-risk participants, 37 met the 2013 ACR/EULAR classification for SSc [19] and 1 participant met the VEDOSS criteria [14]. The median follow-up of the 38 participants and 27 (a subset who developed dcSSc) had similar follow-up 23.8 months (IQR = 11.7–35.5) for all participants vs. 20.7 months (IQR = 5.7–33.9) for those who developed dcSSc, respectively.

**Table 2:** Baseline PRESS demographic and clinical characteristics by final SSc type in the 38 patients in the at-risk population (n = 38)

Baseline characteristics (***n*** = total available data)^∫^	Initial population at high risk of dcSSc at baseline ***N*** = 38	Patients who developed dcSSc during follow-up ***n*** = 27	Patients who did not develop dcSSc during follow-up ***n*** = 11	***P***-value^£^
**Demographic data**				
Age (years), mean (±SD) (*n* = 38)	45.3 (±12.8)	44.4 (±11.9)	47.5 (±14.9)	0.5129^≉^
Gender/female (*n* = 38)	30 (78.9)	23 (85.2)	7 (63.6)	0.1950^§^
Race (*n* = 38)				
Black	4 (10.5)	3 (11.1)	1 (9.1)	1.0000^§^
White	31 (81.6)	21 (77.8)	10 (90.9)	
Others	2 (5.3)	2 (7.4)	0 (0.0)	
Unknown	1 (2.6)	1 (3.7)	0 (0.0)	
Ethnicity (*n* = 38)				
Hispanic	4 (10.5)	4 (14.8)	0 (0.0)	0.3026^§^
Non-Hispanic	34 (89.5)	23 (85.2)	11 (100.0)	
Others/unknown	0 (0.0)	–	–	
Marital status (*n* = 38)				
Single	7 (18.4)	6 (22.2)	1 (9.1)	0.5506^§^
Married	29 (76.3)	20 (74.1)	9 (81.8)	
Divorced or widowed	2 (5.3)	1 (3.7)	1 (9.1)	
Others/unknown	0 (0.0)	–	–	
Employment status (*n* = 38)				
Full-time	25 (65.8)	20 (74.1)	5 (45.5)	0.1109^§^
Part-time	4 (10.5)	1 (3.7)	3 (27.3)	
Retired	3 (7.9)	2 (7.4)	1 (9.1)	
Disability/disabled	0 (0.0)	–	–	
Others^+^	6 (15.8)	4 (14.8)	2 (18.2)	
Smoking status (*n* = 38)				
Never	30 (78.9)	22 (81.5)	8 (72.7)	0.6671^§^
Current or former	8 (21.1)	5 (18.5)	3 (27.3)	
**Clinical data**				
Disease duration (years), mean (±SD), median (IQR)^∫^ since first non-RP symptoms (*n* = 38)	1.0 (±0.5), 0.9 (0.7, 1.3)	1.0 (±0.5), 0.9 (0.5, 1.6)	1.0 (±0.4), 1.0 (0.8, 1.1)	0.5953^€^
Disease duration (years), mean (±SD), median (IQR) since Raynaud’s phenomenon (*n*=36)	3.7 (±6.4), 1.3 (0.8, 2.5)	3.6 (±6.6), 1.8 (0.8, 2.1)	4.0 (±6.2), 1.1 (0.7, 3.6)	0.9589^€^
Disease duration less than 6 months (*n* = 38)	4 (10.5)	4 (14.8)	0 (0.0)	0.3026^§^
First scleroderma symptom (*n* = 38)				
Puffy hands or fingers	19 (50.0)	16 (59.3)	3 (27.3)	0.2152^¶^
Dyspnea	1 (2.6)	0 (0.0)	1 (9.1)	
Arthritis	1 (2.6)	1 (3.7)	0 (0.0)	
Reflux	1 (2.6)	0 (0.0)	1 (9.1)	
Raynaud’s phenomenon	13 (34.2)	8 (29.6)	5 (45.5)	
Skin tightening	1 (2.6)	1 (3.7)	0 (0.0)	
DU	0 (0.0)	–	–	
Others*	2 (5.3)	1 (3.7)	1 (9.1)	
Baseline mRSS (*n* = 34), mean (±SD)	7.4 (±4.8)	8.8 (±4.7)	4.4 (±3.8)	0.0099^≉^
Tendon friction rubs (*n* = 37)	7 (18.9)	7 (26.9)	0 (0.0)	0.0797^§^
Active DU (*n* = 37)	1 (2.7)	1 (3.8)	0 (0.0)	1.0000^§^
Calcinosis (*n* = 35)	4 (11.4)	3 (12.5)	1 (9.1)	1.0000^§^
ILD based on baseline HRCT (*n* = 16)	16 (51.6)	10 (45.5)	6 (66.7)	0.4331^§^
FVC (*n* = 34) (%pred), mean (±SD)	88.6 (±19.0)	86.9 (±19.4)	92.3 (±18.5)	0.4520^≉^
FVC<70% (*n* = 34)	8 (23.5)	5 (21.7)	3 (27.3)	1.0000^§^
DLCO (*n* = 33) (%pred), mean (±SD)	79.7 (±29.8)	80.0 (±26.6)	79.1 (±36.6)	0.9397^≉^
History of PH based on baseline RHC (*n* = 38)	1 (2.6)	1 (3.7)	0 (0.0)	1.0000^§^
Pericardial effusion on first TTE (*n* = 23)	2 (8.7)	2 (14.3)	0 (0.0)	0.5020^§^
LVEF of ≤ 45% on first TTE (*n* = 17)	0 (0.0)	–	–	–
History of Scleroderma renal crisis (*n* = 38)	1 (2.6)	0 (0.0)	1 (9.1)	0.2895^§^
HAQ-DI (n = 34), mean (±SD), median (IQR)	0.7 (±0.6), 0.5 (0.3, 1.0)	0.8 (±0.7), 0.6 (0.4, 1.1)	0.4 (±0.3), 0.3 (0.3, 0.5)	0.0519^€^
**Biological data**				
ANA positive (*n* = 32)	32 (97.0)	23 (95.8)	9 (100.0)	1.0000^§^
Anti-Topo I (*n* = 30)	17 (56.7)	11 (52.4)	6 (66.7)	0.6908^§^
Anti-RNA pol III (*n* = 27)	10 (37.0)	6 (31.6)	4 (50.0)	0.4147^§^
Anti-U3 RNP/fibrillarin (*n*=7)	0 (0.0)	–	–	–
Anti-centromere (*n* = 23)	0 (0.0)	–	–	–
Anti-Th/To (*n* = 8)	4 (50.0)	1 (33.3)	3 (60.0)	1.0000^§^
SSA/anti-RO (*n* = 24)	3 (12.5)	1 (5.9)	2 (28.6)	0.1937^§^
SSB/anti-LA (*n* = 24)	1 (4.2)	1 (5.9)	0 (0.0)	1.0000^§^
Baseline CRP value, mean (±SD), median (IQR) (*n* = 22) mg/dL	2.3 (±2.7), 0.7 (0.4, 4.3)	2.2 (±2.6), 0.7 (0.4, 4.3)	3.1 (±3.4), 1.6 (0.8, 7.0)	0.2305^€^
CRP > ULN (0.6 mg/dL) (*n* = 22)	13 (59.1)	10 (52.6)	3 (100.0)	0.2403^§^

*mRSS*, modified Rodnan skin score; *FVC*, forced vital capacity; *DL*_*CO*_, diffusion capacity for carbon monoxide; *RHC*, right heart catheterization; *LVEF*, left ventricular ejection fraction; *TTE*, transthoracic echocardiogram; *ILD*, interstitial lung disease; *Topo I*, topoisomerase I; *RNA pol III*, RNA polymerase III; *CRP*, C-reactive protein; *HAQ-DI*, Health Assessment Questionnaire-Disability Index; *IQR*, inter-quartile range; *SD*, standard deviation; *ULN*, upper limit of normal

*Other first scleroderma symptom includes lower extremity swelling, telangiectasias, wrist and ankle inflammation, joint pain, fatigue, myalgias, Carpal tunnel syndrome, cold and numbness in extremities, pruritis, hypo/hyper-pigmentation, hypertension, cough, and gastrointestinal discomfort

^∫^Data are expressed as n (%) unless otherwise specified; quantitative data without Gaussian distribution are presented as median (IQR) as specified

^£^Comparison between patients who developed dcSSc during follow-up versus those who did not

^≉^t-test

^¶^Chi-squared test

^§^Fisher exact test

^€^Wilcoxon rank sum test

### Immunosuppressive therapies

At baseline, 63.1% of participants were on any immunosuppressive therapy, of which mycophenolate mofetil (MMF) was most used (40.2%), followed by methotrexate (14.0%) (Table 3). During follow-up, defined as any visit from baseline and onwards, 86.4% of participants were on any immunosuppressive therapy (Table 3). At follow-up, MMF was the most used drug in 68.8% of the participants followed by methotrexate, 21.3%, at any time point during the course of the study (Table 3). 29.9% of participants at baseline and 42.2% of participants at follow-up were on low dose prednisone, with a mean (SD) dose of 9.9 (7.9) mg/day; 9 participants (3.0%) had daily prednisone of >15 mg/ day.

**Table 3:** Immunomodulatory therapies among all PRESS participants at any time during the course of the study

Treatments (n = 301)	Baseline only	Any time during study*
Mycophenolate mofetil, n (%)	121 (40.2)	207 (68.8)
Dose (mg/day), mean (±SD)	1876.9 (±737.0)	2045.4 (±644.5)
Methotrexate, n (%)	42 (14.0)	64 (21.3)
Dose (mg/week), mean (±SD)	14.9 (±6.8)	15.8 (±5.6)
Cyclophosphamide, n (%)	6 (2.0)	15 (5.0)
Dose (mg/day), mean (±SD)	33.6 (±14.4)	44.4 (±27.0)
D-penicillamine, n (%)	5 (1.7)	8 (2.7)
Dose (mg/day), mean (±SD)	650.0 (±285.0)	686.9 (±246.6)
Hydroxychloroquine, n (%)	39 (13.0)	53 (17.6)
Dose (mg/day), mean (±SD)	319.4 (±103.7)	317.8 (±97.1)
Azathioprine, n (%)	5 (1.7)	7 (2.3)
Dose (mg/day), mean (±SD)	115.0 (±41.8)	110.7 (±34.9)
Any immunomodulatory therapy, n (%)	190 (63.1)	260 (86.4)
Autologous hematopoietic stem cell transplantation, n (%)	1 (0.3)	4 (1.3)
Prednisone, n (%)	90 (29.9)	127 (42.2)
Dose (mg/day), mean (±SD)	9.9 (±7.9)	9.2 (±5.2)

*Any time: including all patients with this medication any time during follow-up and/or at baseline

### Skin, internal organ involvement, and association with cancer

**Fig. 1:**
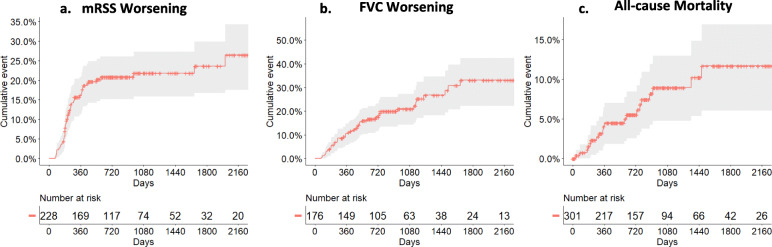
Cumulative skin fibrosis worsening, FVC (%pred) worsening, and all-cause mortality events during the course of the study. **a** Clinically significant worsening of skin fibrosis was defined as an absolute increase of mRSS ≥ 5 units or ≥ 25% as compared to baseline mRSS. **b** Significant functional progression of ILD was defined as an absolute FVC decline of ≥ 10% as compared to baseline FVC. **c** Patients’ vital status was confirmed from medical records or death certificates. mRSS modified Rodnan skin score, FVC forced vital capacity. Gray area corresponds to the 95% confidence interval

Forty-eight participants (21.1%) had a clinical worsening of skin fibrosis (Fig. 1A), and 41 participants (23.3%) had an absolute FVC decline of ≥10% from baseline during the entire course of the study (Fig. 1B). The cumulative incidence of mRSS worsening after 1, 2, and 3 years of follow-up was 19.9%, 20.1%, and 20.3%, respectively. Additionally, the cumulative incidence for FVC worsening after 1, 2, and 3 years of follow-up was 12.7%, 18.3%, and 20.5% (Table 4). Out of 81 participants with ILD who had baseline and follow-up FVC data available, 17 (21.0%) had FVC decline of ≥10% from baseline. TTE data was available on 252 participants (baseline and/or follow-up) and only 8 participants (3.2%) had a LVEF ≤45% (3 participants (1.2%) at baseline, and the remaining 5 (2.0%) during the study). Based on RHC, 7 participants (2.4%) developed PH during follow-up, of which none was PAH, and 11 participants (3.9%) developed SRC during the study (Table 5). In the at-risk group, 12 (45.2%) participants had an absolute increase in mRSS ≥5 units or ≥25%, 7 (30.4%) had an absolute FVC decline of ≥10% from baseline, and 2 (5.4%) developed SRC. None of the participants in the at-risk group had a LVEF of ≤45% on TTE nor PH on RHC (Table 5). The use of immunomodulatory treatments at baseline had no statistically significant impact on the onset of skin progression or FVC decline during follow-up (Table 6) although only 13.6% of the patients remained free of immunomodulatory treatment at the end of follow-up (Table 3).

**Table 4:** Cumulative incidence of events after years of follow-up among all PRESS participants

Outcomes	0–1 year	0–2 years	0–3 years
Overall mortality	11/301 (3.7%)	16/301 (5.3%)	18/301 (6.0%)
mRSS worsening^1,^*	43/216 (19.9%)	45/224 (20.1%)	46/227 (20.3%)
FVC worsening^2,^**	21/165 (12.7%)	32/175 (18.3%)	36/176 (20.5%)

*mRSS*, modified Rodnan skin score; *FVC*, forced vital capacity

^1^Choice of denominator: participants who had baseline mRSS and at least one follow-up mRSS up to n years (where n = 1, 2, or 3) were included in the denominator

^2^Choice of denominator: participants who had baseline FVC% and at least one follow-up FVC% up to n years (where n = 1, 2, or 3) were included in the denominator

*Clinically significant worsening of skin disease was defined as an absolute increase of mRSS ≥ 5 units or ≥ 25% as compared to baseline mRSS

**Significant functional progression of ILD was defined as an absolute FVC decline of ≥ 10% as compared to baseline FVC

**Table 5:** Onset of organ involvement among all PRESS participants during follow-up

Visceral involvement or related outcome measure	Overall population n (%)	Definite dcSSc n/N (%)	At-risk for dcSSc n/N (%)	***P***-value
Absolute increase in mRSS of ≥ 5 units or ≥ 25%, (*n* = 228)^1^	48 (21.1)	36/202 (17.8)	12/26 (45.2)	0.0009^¶^
Absolute decline of FVC % of ≥ 10%, (*n* = 176)^1^	41 (23.3)	34/153 (22.2)	7/23 (30.4)	0.3850^¶^
Pulmonary hypertension on RHC, (*n* = 296)^2, 3,^*	7 (2.4)	7/259 (2.7)	0/37 (0.0)	0.6021^§^
LVEF of ≤ 45% on TTE, (*n* = 189)^3^	5 (2.6)	5/166 (3.0)	0/23 (0.0)	1.0000^§^
Scleroderma renal crisis, (*n* = 285)^3^	11 (3.9)	9/248 (3.6)	2/37 (5.4)	0.6404^§^
All-cause mortality, (*n* = 301)	20 (6.6)	19/263 (7.2)	1/38 (2.6)	0.4870^§^

*mRSS*, modified Rodnan Skin score; *FVC*, forced vital capacity; *RHC*, right heart catheterization; *LVEF*,left ventricular ejection fraction; *TTE*, transthoracic echocardiogram

^1^FVC and mRSS worsening: calculated change from baseline values

^2^mPAP threshold value for pulmonary hypertension was ≥25 mmHg on RHC. Participants who had PH before/at baseline were excluded from the denominator

^3^PH, LVEF, and scleroderma renal crisis: only counted events that occurred between the consent date and the cutoff date

*Based on the results of n = 33 RHC on 29 participants

^¶^Chi-squared test

^§^Fisher exact test

**Table 6:** Impact of baseline immunomodulatory therapies on key outcomes in the PRESS cohort

Progression	Patients with immunomodulatory therapies at baseline	Patients without immunomodulatory therapies at baseline	***P***-value
Patients with skin progression during the entire follow-up	30/142 (21.1%)	18/86 (20.9%)	0.9719
Patient with FVC decline during the entire follow-up	27/114 (23.7%)	14/62 (22.6%)	0.8686
Death at the end of the study	10/190 (5.3%)	10/111 (9.0%)	0.2081

*FVC*, forced vital capacity

Overall, 31 participants (10.3%) had documented cancer, with breast cancer (29%) and non-melanoma skin cancer (19%) being the two most common. Oral, thyroid, and hematological cancers were each found in 10% of participants. Other cancers are listed in Table 7. When considering all types of cancer, with the exception of non-melanoma skin cancer, anti-RNA polymerase III positivity tended to be associated with a previous history or a diagnosis of cancer during follow-up (13 (11.6%) of anti-RNA polymerase III-positive participants with such cancers vs. 6 (5.3%) in the anti-RNA polymerase III-negative participants, p = 0.09) [20]. Seven (6.3%) among the anti-RNA pol III-positive vs. 4 (3.5%) participants had a cancer diagnosed 3 years before or after the first non-RP symptom (p = 0.34).

**Table 7:** Types of cancers among PRESS participants

Type of cancer (***N*** = 31)	N (%)
Breast	9 (29%)
Non-melanoma skin	6 (19%)
Oral	3 (10%)
Thyroid	3 (10%)
Hematological	3 (10%)
Lung cancer	2 (6%)
Melanoma	1 (3%)
Others*	4 (13%)

*Includes esophageal, prostate, uterine, and cervical cancers

### Mortality

The overall cumulative mortality after 1, 2, and 3 years of follow-up was 3.7%, 5.3%, and 6.0%, respectively (Table 4). Overall, 20 participants (6.6%) died (Table 8), of which 18 (90%) were attributed as SSc-related deaths. The two most common causes of SSc-related deaths were severe gastrointestinal dysmotility (22.2%) and cardiac involvement (33.3%; including cardiac arrhythmia (22.2%), cardiac arrest and seizures (5.6%), and congestive heart failure (5.6%)) (Table 8). Three patients (16.7%) died from ILD, which was the third cause of SSc-related death. One participant (2.6%) from the at-risk group died from progressive ILD. Other causes are listed in Table 8. Patients with anti-RNA pol III antibodies, the main represented antibody subtype in the overall PRESS cohort, did not differ in terms of survival as compared to patients with other antibody subtypes considered altogether (p = 0.973; data not shown). Patients with baseline immunomodulatory treatment tended to have a lower mortality at the end of follow-up although this result was not statistically significant (mortality of 5.3% at the end of the study in patients with baseline immunomodulatory agents versus 9.0% in the group without baseline immunomodulatory therapies, p = 0.21; Table 6).

**Table 8:** Cause of death among PRESS participants who died during the course of the study

Cause of death (n = 20)	n (%)
**SSc related**	18 (90.0)
**Cardiac**	
Cardiac arrhythmia	4 (22.2)^+^
Cardiac arrest and seizures	1 (5.6)^+^
Congestive heart failure	1 (5.6)^+^
**Gastrointestinal**	
Severe GI dysmotility	4 (22.2)^+^
**Pulmonary**	
Progressive ILD	3 (16.7)^+^
**Cardiopulmonary**	
Significant PAH	1 (5.6)^+^
**Renal**	
Scleroderma renal crisis	1 (5.6)^+^
**Multi-systemic**	
Scleroderma renal crisis, severe GI dysmotility, and severe PH	1 (5.6)^+^
Acute hypoxemic failure, cardiogenic shock due to probable PE	1 (5.6)^+^
**Others**	
Cardiac toxicity due to CYC	1 (5.6)^+^
**Non-SSc related**	2 (10.0)
**Esophageal cancer**	2 (100)^++^

*ILD*, interstitial lung disease; *GI*, gastrointestinal; *PAH*, pulmonary arterial hypertension; *PH*, pulmonary arterial hypertension; *PE*, pulmonary embolism; *CYC*, cyclophosphamide

^+^Percentage based on SSc-related death

^++^Percentage based on non-SSc-related death

## Discussion

dcSSc has one of the highest case fatality rates in rheumatic diseases [1]. With recommendations from different societies advocating yearly screening and early diagnosis for internal organ involvement in SSc [14] and increased use of immunosuppressive therapies for management of early SSc, we sought to comprehensively assess the outcomes in this cohort in the current era. In this early at-risk or dcSSc registry, 86% of the patients were on immunosuppressive therapy during the course of the study and MMF was the most frequently prescribed medication. Despite this, there was worsening of the skin in approximately 20% of patients and continuing decline in FVC in almost 20% of patients. The overall mortality was 6.6% during a median follow-up of 24.5 months, with cardiac and gastrointestinal involvement as the leading causes of mortality.

In comparison to other dcSSc cohorts, patients included in PRESS and in the University of Pittsburgh cohort had similar disease duration [13]. Considering baseline data in definite dcSSc, patients in PRESS (n = 263) had somewhat less severe disease for baseline prevalence of SRC (18% in Pittsburgh derivation cohort versus 5.7% in PRESS [12]), TFR (59% versus 36.3% in Pittsburgh and PRESS, respectively [12]) and mean baseline mRSS (26.8 (±11.9) and 22.9 (±9.3) in Pittsburgh and PRESS, respectively). The University of Pittsburgh is a referral center, which might reflect a selection bias of more severe disease. An earlier or broader use of immunomodulatory drugs as well as increased education about avoiding high dose steroids and regular home blood pressure monitoring in the past 20 years may also have contributed to limit the prevalence of SRC in PRESS in comparison with the historical US cohorts from Pittsburgh (inclusion period 1980–2007). The current baseline data in PRESS confirms that patients in the US with dcSSc tend to have a higher prevalence of musculoskeletal and renal involvement in comparison with European patients, since TFR prevalence ranged from 12.8 to 20.2% [21, 22] in EUSTAR and SRC prevalence was under 5% in recent publications from EUSTAR and ESOS (European Scleroderma Observational Study) [21, 23]. This lower prevalence of SRC in European cohorts could be explained by the lower prevalence of anti-RNA polymerase III antibodies in these dcSSc European cohorts (positivity of RNA polymerase III in 8.4 to 19.1%) in comparison with existing US cohorts (positivity of RNA polymerase III in 34.2 to 63%) [9, 21, 23–26]. Mean baseline mRSS in definite dcSSc from PRESS was also higher than in European studies (median mRSS 16 (IQR 11–23) in EUSTAR, 21 (IQR 16–27) in ESOS) [21, 23]. This difference could be explained by the higher prevalence of RNA polymerase III antibodies in PRESS as these antibodies are associated with a higher peak of mRSS [27]. Mean mRSS in PRESS is consistent with the results from the Australian or US GENISOS registries [9, 28] and with baseline characteristics of patients with early dcSSc in recent RCTs [29–31].

When assessing for internal organ involvement at baseline, the prevalence of ILD on HRCT in the overall PRESS cohort (53.6%) is consistent with recent publications of EUSTAR (57% with ILD [21, 32]) and more frequent than in the Pittsburgh and ESOS cohorts (27% and 14.4%, respectively [12, 23]). Broader use of HRCT in PRESS and EUSTAR may explain this result since the presence of pulmonary involvement could rely on X-rays only and not systematic HRCT in the Pittsburgh and ESOS cohorts [15]. In the focuSSced trial, approximately 66% of the patients had ILD based on baseline HRCT evaluation. This prevalence was higher than in PRESS and this could be explained by uniform HRCT performed in every patient and the specific selection of patients with elevated acute-phase reactant levels and active skin disease [29, 33]. Considering cardiac involvement, 13.9% of the patients from PRESS had pericardial effusion on TTE which is consistent with prevalence from previous studies [34]. Eight patients (3.2%) had LVEF <45% at baseline or during follow-up. TTE alone does not allow us to infer that the decrease of LVEF was directly linked to scleroderma and MRI evaluation is warranted to determine the precise cause of LVEF dysfunction. The issue of early cardiac involvement, and the prognostic value of specific TTE features in early dcSSc is still to be further explored, as well as the precise definition of SSc-related cardiac involvement beyond PAH.

In PRESS, the use of immunosuppressive drugs was reported in 63.1% of the patients at baseline, 85.7% during the first year, and 86.4% at any time. This result is similar in EUSTAR and ESOS but higher than in the US GENISOS cohort with less than 56% of immunosuppressive drugs at baseline and patients’ inclusion period starting in 1998. Immunosuppressive drugs were also more frequent in PRESS than in national registries such as the German network (inclusions from 2003 to 2007) reported immunosuppressive drugs in 46.4% of patients with dcSSc [35]. Immunosuppressive therapies were also less frequently used in the Canadian registry (less than 40% of dcSSc) than in PRESS [36]. These results may reflect a general trend toward broader and earlier use of immunosuppressive drugs in the current era for dcSSc patients in the US. MMF was the most frequently prescribed immunosuppressive drug in PRESS (68.8% of all patients). This may reflect the incorporation of results of SLS II, a US-based study, by the physicians in the PRESS registry. Prednisone was used in 42.2% any time during the study, and this frequency was similar to the German Network study [35]. This result on steroid use is also consistent with baseline data from recent RCTs including early dcSSc patients [30]. Five patients underwent stem cell transplantation (SCT) in the PRESS registry (1.6%) suggesting that this therapeutic approach is still rarely used in dcSSc patients in the US despite the encouraging results of the SCOT trial [37].

Our results confirm the overall high progressive trajectory for the worsening of mRSS, particularly in the first year of follow-up [32], whereas FVC showed a more progressive decrease during the three first years. In accordance with previous studies, this result demonstrates the relevance of including early dcSSc patients for RCTs based on mRSS evolution [21, 30, 38, 39]. In PRESS, approximately 20% of the patients had significant mRSS worsening after 1 year of follow-up. This is greater than the 10% reported in EUSTAR [8] and 11.2% in GENISOS [9]. This could be explained by a shorter baseline disease duration in PRESS or by a higher prevalence of anti-RNA polymerase III antibodies, as they are associated with earlier diffuse cutaneous involvement [27, 40]. This result supports a stratification on antibody subtypes in RCTs with mRSS as the primary outcome. FVC decrease was similar in PRESS and EUSTAR (almost 13% experienced a decrease of 10% of FVC after 1 year of follow-up in both cohorts) with a similar prevalence of ILD on HRCT [32]. This rate of progression was also similar in the placebo arm from the intention to treat population of the focuSSed trial (17% at week 48) [29]. Based on clinically meaningful definitions of progressors, only half of FVC progressors were detected within the first year of follow-up in PRESS. This result may suggest that cumulative damage cannot be properly captured over 1 year of follow-up, particularly in patients receiving baseline immunosuppressive drugs, and that longer follow-up duration should be discussed for RTCs [41]. Our work also confirms a trend toward an association of anti-RNA polymerase III antibodies and cancers although the limited follow-up and the small number of patients with cancer limited statistical significance to confirm the association of SSc and synchronous cancer in patients with anti-RNA polymerase III antibody.

The overall mortality rate in PRESS was lower than 10% after a mean follow-up of 746 days, with a first-year mortality rate of less than 5% and a 2-year mortality rate of less than 6%. This first-year mortality rate is lower than in a recent EUSTAR study with 13% of death after 12 (±3) months of follow-up [42]. The 2-year mortality rate in PRESS is also lower than that in the Pittsburgh derivation cohort (22%) and in their internal validation cohort (12%) but similar to previous European registries [12, 43, 44]. Although it is difficult to be definitive without a carefully planned prospective cohort/trial, our results may demonstrate that broader use of immunosuppressive therapies and lower doses of steroids may have led to lower mortality rate, as well as broader use of ACE inhibitors for SRC. A 3-year mortality rate of 6% is still high and a disease-modifying drug that could simultaneously target multiple visceral damages to provide an overall improvement of the disease and limit mortality is thus needed [45]. In our study, cardiac (33.3%) and gastrointestinal involvement (22.2%) were the leading causes of death, with 16.7% of death due to SSc-ILD. This result differs from the EUSTAR and Canadian registry where ILD was a major cause of death [46, 47]. Low prevalence of anti-topoisomerase I in PRESS by comparison with EUSTAR may explain these differences [48]. ILD-related death may also occur more lately in the course of the disease, and a longer follow-up duration is needed to confirm our results. Another explanation could be the positive impact of earlier MMF introduction on FVC evolution [5], confirming the trend toward global improvement in the management of early dcSSc in the last decade.

This PRESS study also explored patients with limited skin involvement at baseline but at high risk of subsequent evolution to dcSSc. Most of these patients at-risk of dcSSc at baseline developed diffuse cutaneous involvement during follow-up (27/38 (71.1%)), demonstrating the relevance of the “at-risk” inclusion criteria. Higher mRSS at baseline was associated with the development of dcSSc within this at-risk subgroup. Similarly, TFR is a risk factor for future onset of dcSSc, as all the patients from the at-risk group who had TFR at baseline developed dcSSc during follow-up. This result on TFR is concordant with previous results from the Pittsburgh cohort [49]. Patients who developed dcSSc also tended to have higher baseline HAQ-DI (P = 0.052), suggesting that initial overall severity may be a risk factor for dcSSc among at-risk patients. No other baseline characteristics, including autoantibody subtypes, differentiated patients that developed dcSSc from those who did not among the baseline non-dcSSc, but the small sample size (38 patients) precludes conclusions. Interestingly, almost 50% of the patients from both groups (at-risk and definite) had puffy fingers or puffy hands as the first scleroderma-associated manifestation, whereas RP was the first manifestation in only 21.3% of the overall PRESS population. This result is similar from a recent publication from the Pittsburgh group where RP was the first scleroderma-associated symptom in only 28% of early dcSSc patients [50]. This highlights the relevance of puffy hands/fingers as a criterion for the very early diagnosis of systemic sclerosis (VEDOSS) [51].

The strengths of this study are inclusions of early disease dcSSc patients in comparisons with previous multicenter cohorts, its nationwide scale, and prospective follow-up allowing a precise standardized analysis and deep phenotyping of patients by well-trained physicians. The recruitment rate in the different centers was not recorded in this registry started in 2012 and this is one of the limitations of this study. We were thus not able to specify among the patients seen during the inclusion period and fulfilling the selection criteria which proportion was finally recruited in the PRESS registry. This limits the discussion of a potential selection bias. The limitations of this study also include the limited follow-up duration as the registry is ongoing and we will learn more about the internal organ involvement and related mortality in longer term follow-up. This absence of long-term data may have led to a low event rate that precludes sub-analysis and specific survival modeling. Longer follow-up duration of this cohort may also help to confirm the importance of antibody status and their relevance in association with molecular signatures for patients’ stratification and prediction of skin trajectory or organ involvement [52]. This is a major issue as the improvement of patient selection is a key aspect for the design of clinical trials in early dcSSc [53].

## Conclusion

This study based on the PRESS registry is the largest multicenter US study assessing baseline characteristics, treatment patterns, and disease progression in patients with early at-risk or dcSSc in the current era. Our results highlight the very early progression of skin involvement in this cohort including a high proportion of patients with RNA pol III antibodies. The 3-year mortality rate of 6% despite early use of immunosuppressive therapies demonstrates the unmet need for disease-modifying drugs in dcSSc and highlights that efforts are needed to foster RCTs dedicated to this subset of SSc [45, 53]. Careful monitoring of very early SSc with limited cutaneous involvement but at high risk of developing dcSSc based on the presence of swollen hands or sclerodactyly associated with anti-topoisomerase I or anti-RNA polymerase III antibodies, and/or presence of tendon friction rubs, may help to improve the early management of dcSSc and may be considered for the design of future RCTs.

## Data Availability

The datasets used and/or analyzed during the current study are available from the corresponding author on reasonable request.

## Abbreviations

ACR/EULAR: American College of Rheumatology/European League Against Rheumatisms
dcSSc: Diffuse cutaneous systemic sclerosis
EUSTAR: European Scleroderma Trials and Research group
FVC: Forced vital capacity
ILD: Interstitial lung disease
lcSSc: Limited cutaneous systemic sclerosis
LVEF: Left ventricular ejection fraction
MMF: Mycophenolate mofetil
mPAP: Mean pulmonary arterial pressure
mRSS: Modified Rodnan skin score
PAH: Pulmonary arterial hypertension
PFT: Pulmonary function test
PH: Pulmonary hypertension
PRESS: Prospective Registry of Early Systemic Sclerosis
RCTs: Randomized control trials
RHC: Right heart catheterization
RP: Raynaud’s phenomenon
SD: Standard deviation
SRC: Scleroderma renal crisis
SSc: Systemic sclerosis
TFR: Tendon friction rubs
TTE: Transthoracic echocardiogram
US: United States
VEDOSS: Very early diagnosis of systemic sclerosis
